# A Statistical Test for Differential Network Analysis Based on Inference of Gaussian Graphical Model

**DOI:** 10.1038/s41598-019-47362-7

**Published:** 2019-07-26

**Authors:** Hao He, Shaolong Cao, Ji-gang Zhang, Hui Shen, Yu-Ping Wang, Hong-wen Deng

**Affiliations:** 1 Center for Bioinformatics and Genomics, Department of Global Biostatistics and Data Science, Tulane University School of Public Health and Tropical Medicine, New Orleans, LA 70112 USA; 2 Department of Biomedical Engineering, Tulane University, New Orleans, LA 70118 USA

**Keywords:** Computational models, Network topology

## Abstract

Differential network analysis investigates how the network of connected genes changes from one condition to another and has become a prevalent tool to provide a deeper and more comprehensive understanding of the molecular etiology of complex diseases. Based on the asymptotically normal estimation of large Gaussian graphical model (GGM) in the high-dimensional setting, we developed a computationally efficient test for differential network analysis through testing the equality of two precision matrices, which summarize the conditional dependence network structures of the genes. Additionally, we applied a multiple testing procedure to infer the differential network structure with false discovery rate (FDR) control. Through extensive simulation studies with different combinations of parameters including sample size, number of vertices, level of heterogeneity and graph structure, we demonstrated that our method performed much better than the current available methods in terms of accuracy and computational time. In real data analysis on lung adenocarcinoma, we revealed a differential network with 3503 nodes and 2550 edges, which consisted of 50 clusters with an FDR threshold at 0.05. Many of the top gene pairs in the differential network have been reported relevant to human cancers. Our method represents a powerful tool of network analysis for high-dimensional biological data.

## Introduction

It is well-acknowledged that a complex disease is rarely a consequence of an abnormality of a single gene product, but involves various pathological processes that interact in a complex network^1^. The better understanding of the effects of molecular and cellular network in disease etiology has multiple potential biological and clinical applications. It will help identify pivotal disease risk genes and pathways and provide better targets for drug development.

Previous methods for network analysis mainly focused on correlation-based metrics to measure the strength of association between gene pairs in a network^2–4^. However, these methods, which only explore marginal correlations, cannot distinguish direct or indirect relationships between genes. Gaussian graphical model (GGM) is a relatively more realistic way to present complex network because of its interpretation with conditional dependence between two variables after removing the effects of all other variables^5^. GGM can filter out all high correlations which are attributed to other genes, and also can lead to genes highly related in terms of partial correlations with other neighboring genes^5^. GGMs are closely linked to precision matrices, which describe the graphical structure of the corresponding Gaussian graph. It is a great challenge to construct biological networks through GGM in high-dimensional setting, in which the number of variables or features is much larger than the sample size. The basic idea behind it is that high-dimensional biological data are sparse in the sense that only a small number of genes will regulate one specific gene of interest^5^. This scenario leads to the construction of an undirected graph of conditional dependencies which is sparser than a correlation network^5^.

During the last decade, many methods for estimating GGM in the high-dimensional settings have been developed based on certain sparseness assumptions. One of the most widely used methods was the graphical Lasso (GLasso) method through the use of *L*_1_ (lasso) regularization^6^. Cai *et al*. developed a constrained *L*_1_ minimization approach to estimate sparse precision matrix^7^. More recently, Ren *et al*. proposed a novel method to obtain asymptotically normal and efficient estimation of large GGM under a minimal sparseness condition^8^, which is the first theoretical study to estimate partial correlations as well as p-value and confidence interval for each edge in the graph. In addition, a fast algorithm, named “FastGGM^9^”, as an exact implementation to the asymptotically normal and efficient estimation established by Ren *et al*.^8^, showed that the inference of partial correlation between genes becomes computationally feasible for whole-genome data sets^9^. All of these methods addressed the problem of estimating and constructing a single Gaussian graphical model.

Differential network analysis, which investigates how the network of connected genes changes from one condition to another, has become a prevalent tool to provide a deeper and more comprehensive understanding of complex diseases^10^. Several recent studies have demonstrated the power of differential network analysis for elucidating fundamental and key biological responses, revealing that the architecture of gene network can be rewired during a cellular or adaptive response^10–12^. It is of great biological interest in many applications to estimate the precision matrices and the corresponding graphical structures over different groups or conditions. A differential network between two groups can be constructed by the difference between the two precision matrices, which is interpreted as the differences in the partial covariances of each pair of genes between the two groups. It’s notable that the gene network in different groups are often similar to each other, the graphical structures would share many common edges. In the differential network, the significant connections are discovered to differentiate from one condition to the other while weak and common ones are removed^10^. A joint graphical lasso (JGL) has been proposed to preserve the common graphical structure while allowing differences across groups^13^. This method is based on maximizing a penalized log likelihood with a fussed Lasso or group Lasso penalty. However, there was no theoretical justification on the statistical convergence rate of the estimators in the method and the results were heavily dependent on the choice of tuning parameters. Recently, a pathway-based differential network analysis model (DINGO: Differential Network Analysis in Genomics) has been developed to jointly estimate the group-specific conditional dependencies by decomposing them into global and group-specific components^14^. However, the computational time involved in model fitting made it impractical to handle more than 2000 genes. Moreover, these approaches assumed that precision matrices in different groups were sparse without considering the structure of real gene network. For example, real regulatory gene network often contains hub nodes, therefore the rows and columns of precision matrix corresponding to hub nodes have many nonzero entries, possibly violating the sparsity condition^15^.

In the present study, through the GGM framework, we developed a computationally efficient test to infer the differential network structure through testing the equality of two precision matrices in the high-dimensional setting and applied a multiple testing procedure with FDR control. We evaluate our method and compare it with other estimation approaches via simulations under different parameter settings. Then we applied our method to a lung cancer dataset. Using both simulated and real data sets, we demonstrate that our method is a powerful tool for differential network analysis in high-dimensional biological data.

## Methods

### Notation and basic model

Let ${\bf{X}}={[{{\bf{x}}}^{1},\ldots ,{{\bf{x}}}^{{n}_{1}}]}^{T}\in {R}^{{n}_{1}\times p}$ and ${\bf{Y}}={[{{\bf{y}}}^{1},\ldots ,{{\bf{y}}}^{{n}_{2}}]}^{T}\in {R}^{{n}_{2}\times p}$, denote the data matrices. Note that {${{\rm{x}}}^{1},\ldots ,{{\rm{x}}}^{{n}_{1}}$} and {${{\rm{y}}}^{1},\ldots ,{{\rm{y}}}^{{n}_{2}}$} are independent observations from two populations. Assume that ${{\bf{x}}}^{i}={[{x}_{1}^{i},\cdots {x}_{P}^{i}]}^{T}$ and ${{\bf{y}}}^{j}={[{y}_{1}^{j},\cdots {y}_{P}^{j}]}^{T}$ for *i*th and *j*th individual is an independently and identically distributed sample from a Gaussian distribution $N({0}_{P},{\Sigma }_{1})$ and $N({0}_{P},{\Sigma }_{2})$, respectively, where 0_*P*_ is a vector of *P* **0**’s and ∑_1_ and ∑_2_ is a *P* × *P* covariance matrix. Let ${{\Omega }}_{d}=({\omega }_{ij,d})={{\sum }^{}}_{d}^{-1}$ for *d* = 1, 2 be the precision matrix for X and Y, respectively.

It is known that the precision matrix (inverse covariance matrix) *Ω* = Σ^−1^ represents a GGM, where the non-zero (or zero) value for *ω*_*k*,*l*_ in the (*k*, *l*)th entry of Ω represents the presence (or absence) of edges between *k*th and *l*th variable. A GGM associated with **X** is a graph, where the node set V = {**x**_1_, **x**_2_, …, **x**_*p*_} has *p* components and the edge set E such that any edge between **x**_*k*_ and **x**_*l*_ if and only if **x**_*k*_ and **x**_*l*_ are conditional dependent given all other variables. Similarly, a GGM associated with **Y** is also a graph. The methodology of the present study is based on GGM and translates the differential network analysis with a binary trait D into the statistical inference and comparison of two high-dimensional precision matrices.

### Inference of GGM

The problems of estimating a large sparse precision matrix have drawn considerable recent attention. Recently, Ren *et al*. and Sun *et al*. made important advancements in the statistical inference in the GGM^8,16^. Especially, Ren *et al*. proposed an adaptive estimator of individual *ω*_*ij*_ and proved its asymptotic normality and efficiency under the sparseness assumption of the graph^8^. The efficient estimator of the individual *ω*_*ij*_ was then used to construct fully data-driven procedures to recover the support of and to make statistical inference about latent variables in the graphical model.

Briefly, consider an index set *A* = {*i*,*j*} with *i* ≠  *j*. In the Gaussian setting the precision matrix can be described in terms of regression models. For *n*_1_ × *p* data matrix **X**, we regress the *i*th and the *j*th columns **X**_**A**_ against the remaining columns ${{\boldsymbol{X}}}_{{{\boldsymbol{A}}}^{{\boldsymbol{c}}}}$. Specifically, we may write ${{\boldsymbol{X}}}_{{\boldsymbol{A}}}={{\boldsymbol{X}}}_{{{\boldsymbol{A}}}^{{\boldsymbol{c}}}}{\boldsymbol{\beta }}+{\epsilon }_{{\boldsymbol{A}}}$, where the true coefficients ${\boldsymbol{\beta }}={{\boldsymbol{\beta }}}_{{{\boldsymbol{A}}}^{{\boldsymbol{c}}},{\boldsymbol{A}}}=-{{\Omega }}_{{A}^{c},A}{{\Omega }}_{A,A}^{-1}$ and rows of ${\epsilon }_{{\boldsymbol{A}}}$ are i.i.d. Gaussian vectors with mean zero mean and covariance ${{\Omega }}_{A,A}^{-1}$. Scaled Lasso was used for the regression to obtain the estimator $\hat{{\boldsymbol{\beta }}}$ of ***β*** and the residual ${\hat{\epsilon }}_{{\boldsymbol{A}}}={{\boldsymbol{X}}}_{{\boldsymbol{A}}}-{{\boldsymbol{X}}}_{{{\boldsymbol{A}}}^{{\boldsymbol{c}}}}\hat{{\boldsymbol{\beta }}}$. Therefore, for **X** the estimated precision matrix $${\hat{{\boldsymbol{\Omega }}}}_{{\boldsymbol{A}},{\boldsymbol{A}}}=(\begin{array}{cc}{\hat{\omega }}_{ii,} & {\hat{\omega }}_{ij}\\ {\hat{\omega }}_{ij} & {\hat{\omega }}_{jj}\end{array})={(\frac{1}{n}{\hat{\epsilon }^{\prime} }_{{\boldsymbol{A}}}{\hat{\epsilon }}_{{\boldsymbol{A}}})}^{-1}$$. Note that scaled Lasso provides scale-free simultaneous estimation of the regression coefficients and noise level. It is a tuning-free penalized approach so that it can avoid the cross-validation procedures^8^. The estimator ${\hat{\omega }}_{ij}$ is asymptotically efficient,

1 $$\sqrt{n{\hat{F}}_{ij}}({\hat{\omega }}_{ij}-{\omega }_{ij})\mathop{\to }\limits^{{\rm{D}}}N(0,1),$$

where ${\hat{F}}_{ij}={({\hat{\omega }}_{ii}{\hat{\omega }}_{jj}+{\hat{\omega }}_{jj}^{2})}^{-1}$. ${\hat{F}}_{ij}$ is the estimator of *F*_*ij*_, which is the Fisher information for estimating *ω*_*ij*_. The lower bound is established through Le Cam’s lemma^8^. Partial correlation, which is used to measure the strength of conditional dependence, is calculated as ${\hat{\gamma }}_{ij}=-{\hat{\omega }}_{ij}/\sqrt{{\hat{\omega }}_{ii}{\hat{\omega }}_{jj}}$ with the property

2 $$\sqrt{n{(1-{\hat{\gamma }}_{ij}^{2})}^{-2}}({\hat{\gamma }}_{ij}-{\gamma }_{ij})\mathop{\to }\limits^{{\rm{D}}}N(0,1).$$

It is worthwhile to point out that the asymptotic efficiency result is obtained without the need to assume the irrepresentability condition or the *L*_1_ constraint of the precision matrix which are commonly required in the literature.

### Hypothesis testing of differential networks

Let $${\bf{X}}={[{{\bf{x}}}^{1},\ldots ,{{\bf{x}}}^{{n}_{1}}]}^{T}\in {R}^{{n}_{1}\times p}$$ and $${\bf{Y}}={[{{\bf{y}}}^{1},\ldots ,{{\bf{y}}}^{{n}_{2}}]}^{T}\in $$$${R}^{{n}_{2}\times p}$$, denote the data matrices. Note that {$${{\rm{x}}}^{1},\ldots ,{{\rm{x}}}^{{n}_{1}}$$} and {$${{\rm{y}}}^{1},\ldots ,{{\rm{y}}}^{{n}_{2}}$$} are independent observations from two populations. Let $${{\Omega }}_{d}=({\omega }_{ij,d})={{\sum }^{}}_{d}^{-1}$$ for *d* = 1, 2 be the precision matrix for X andY, respectively. The difference between two precision matrices from **X** and **Y**, respectively, is called the differential network and denoted by $${\rm{\Delta }}={\delta }_{ij}={{\Omega }}_{1}-{{\Omega }}_{2}={\omega }_{ij,1}-{\omega }_{ij,2}$$. We aim to make statistical inference of each edge in the differential network, or equivalently of each *δ*_*ij*_ by testing the hypothesis

3 $${{\boldsymbol{H}}}_{0,ij}:{w}_{ij,1}-{w}_{ij,2}=0\,{\rm{versus}}\,{{\boldsymbol{H}}}_{1,ij}:{w}_{ij,1}-{w}_{ij,2}\ne 0,\,1\le i\le j\le p$$

Based on the inference of GGM and asymptotic normality of *ω*_*ij*_, we derive the test statistics as

$${W}_{ij}=\frac{{\hat{\omega }}_{ij,1}-{\hat{\omega }}_{ij,2}}{\sqrt{\frac{{\hat{\omega }}_{ii,1}{\hat{\omega }}_{jj,1}+{\hat{\omega }}_{jj,1}^{2}}{{n}_{1}}+\frac{{\hat{\omega }}_{ii,2}{\hat{\omega }}_{jj,2}+{\hat{\omega }}_{jj,2}^{2}}{{n}_{2}}}}$$

Shown in the section of Inference of GGM, as the network graph is sufficiently sparse, the estimator ${\hat{{\rm{\omega }}}}_{{\rm{ij}}}\,$ is asymptotically efficient in the sense that $\sqrt{n{\hat{F}}_{ij}}({\hat{\omega }}_{ij}-{\omega }_{ij})\mathop{\to }\limits^{{\rm{D}}}N(0,1)$. where ${\hat{F}}_{ij}={({\hat{\omega }}_{ii}{\hat{\omega }}_{jj}+{\hat{\omega }}_{jj}^{2})}^{-1}$. ${\hat{F}}_{ij}$ is the estimator of *F*_*ij*_, which is the Fisher information for estimating *ω*_*ij*_. Equivalently, the asymptotic normality of the estimator ${\hat{\omega }}_{ij}$ has mean *ω*_*ij*_ and variance ${n}^{-1}({\omega }_{ii}{\omega }_{jj}+{\omega }_{ij}^{2})$. Note that {${{\rm{x}}}^{1},\ldots ,{{\rm{x}}}^{{n}_{1}}$} and {${{\rm{y}}}^{1},\ldots ,{{\rm{y}}}^{{n}_{2}}$} are independent observations from two populations. *ω*_*ij*,*d*_ for *d* = 1, 2 be the precision matrix for X and Y, respectively. The estimator ${\hat{{\rm{\omega }}}}_{{\rm{ij}},{\rm{d}}}$ is asymptotically efficient that

$$\sqrt{n{\hat{F}}_{ij,d}}({\hat{\omega }}_{ij,d}-{\omega }_{ij,d})\mathop{\to }\limits^{{\rm{D}}}N(0,1).$$

where ${\hat{F}}_{ij,d}={({\hat{\omega }}_{ii,d}{\hat{\omega }}_{jj,d}+{\hat{\omega }}_{jj,d}^{2})}^{-1}$. Intuitively, ${\hat{\omega }}_{ij,1}-{\hat{\omega }}_{ij,2} \sim N$(${\omega }_{ij,1}-{\omega }_{ij,2},$[${n}_{1}^{-1}({\omega }_{ii,1}{\omega }_{jj,1}+{\omega }_{ij,1}^{2})+$ ${n}_{2}^{-1}({\omega }_{ii,2}{\omega }_{jj,2}+{\omega }_{ij,2}^{2})$]). Under the null ***H***_0,***ij***_ variables {*W*_*ij*_} would follow standard normal distributions.

### Multiple testing with false discovery rate (FDR) control

For testing *p*(*p* − 1)/2 hypotheses, multiple testing procedures using Bonferroni correction or naive false discovery rate corrections may lose power. In order to carry out simultaneous testing on the structure of the differential network Δ with FDR control, we used the following multiple testing procedure^17^.

1. For a given pre-specified level α, calculate $\hat{t}={\rm{\inf }}\{0\le t\le 2{(\mathrm{log}p)}^{\frac{1}{2}}\frac{2\{1-\phi (t)\}({p}^{2}-p)/2}{R(t){\rm{v}}1}\le \alpha \}$. If $\hat{t}$ does not exist, set $\hat{t}=2{(\mathrm{log}p)}^{\frac{1}{2}}$.
2. For1 ≤ *i* ≤ *j* ≤ *p*, reject ***H***_0,***ij***_ if and only if $|{W}_{ij}|\ge \hat{t}$.

Note that *t* is the threshold level such that ***H***_0,***ij***_ is rejected if |*W*_*ij*_| ≥ *t*. *φ*(*t*) is a standard normal cumulative distribution function and $R(t)={\sum }_{1\le i\le j\le p}I(|{W}_{ij}|\ge t)$ denotes the total number of rejections^17^. The ideal choice of *t* would reject as many true positives as possible in the meantime of controlling the FDR at the given pre-specified level α.

### Simulation study

To evaluate the performance of our method, we designed realistic simulations. In the setting of n < p, we simulated two group data from multivariate normal distributions with different undirected graph structures, including hub, scale-free, and random graphs, in which some of the edges are common to both groups.

Suppose we had two groups *d* = 1 and 2, respectively. First, a common undirected graph structure was simulated for the two groups. A precision matrix *Ω* was generated using *huge*.*generator* in the R package Huge^18^. As the common graph, we considered three different graph structures, including hub, scale-free, and random graph. The off-diagonal elements of the precision matrix were set to be 0.5 and a positive number added to the diagonal elements of the precision matrix was 0.2. The number of hubs in the hub graph and the probability that a pair of nodes had an edge in the random graph were default values set in the function *huge*.*generator*^18^. The scale-free graph was generated using Barabasi-Albert algorithm implemented in the function *huge*.*generator*^18,19^. Second, set *Ω*_1_ = *Ω*_2_ = *Ω* and replace a randomly chosen entry from the zero entries in *Ω*_1_ and *Ω*_2_ with a uniform random sample. Third, repeat the second step γ × M times, where M is the number of nonzero entries in *Ω* and γ is the heterogeneity of the graphs, which is used to control the ratio of the number of individual edges to the number of common edges. Fourth, generate **x**^*i*^ and **y**^*i*^ for *i*th individual in two groups from the distributions $N({0}_{P},{{\Omega }}_{1}^{-1})$ and $N({0}_{P},{{\Omega }}_{2}^{-1})$, respectively. A workflow of simulation steps was shown in Supplementary Fig S1 and the details of the simulation settings were shown in the Table 1. In each scenario, the datasets were simulated with 100 replications based on four parameters including the sample size *n*, the number of vertices *p*, the level of heterogeneity *γ* and graph structure.

**Table 1:** Comparison of estimating the group specific precision matrix and differential network.

Sample size *n*_1_ = *n*_2_	Number of vertices *p*	Level of heterogeneity *γ*	Graph structure	Our method^a^	DINGO^a^	JGL^b^	Our method^b^	DINGO^b^
50	100	0.25	Random	0.832	0.718	0.508	0.643	0.522
			Hub	0.844	0.807	0.521	0.632	0.521
			Scale-free	0.777	0.644	0.505	0.640	0.514
50	100	0.75	Random	0.815	0.708	0.532	0.660	0.516
			Hub	0.799	0.727	0.563	0.640	0.513
			Scale-free	0.769	0.616	0.510	0.653	0.519
100	200	0.25	Random	0.828	0.657	0.505	0.648	0.515
			Hub	0.868	0.799	0.527	0.637	0.513
			Scale-free	0.734	0.592	0.502	0.645	0.505
100	200	0.75	Random	0.835	0.680	0.537	0.677	0.513
			Hub	0.832	0.740	0.595	0.658	0.515
			Scale-free	0.756	0.580	0.510	0.665	0.518

Note: JGL, joint graphical lasso; DINGO, Differential Network Analysis in Genomics.

^a^AUC values group specific precision matrix.

^b^AUC values for differential network.

## Results

Note that Ren *et al*. had reported better performance of the tuning-free inference methodology than the existing *L*_1_ penalized methods in the estimation of the precision matrix^8,9^. Additionally, we compared its performance with JGL and DINGO in scenarios of different undirected graph structures, in which some of the edges are common to both groups. We evaluated the estimation of nonzero elements in true precision matrices *Ω*_1_ and *Ω*_2_, as well as in true Δ. Receiver Operating Characteristic (ROC) curve was generated using the estimated p-values and the true zero/nonzero elements in the precision matrix. And then the Area Under the Curve (AUC) was averaged over 100 replications to measure the performance of the estimation. For JGL, there were two tuning parameters. We first chose optimal tuning parameters by Bayesian information criterion (BIC) and then calculated the averaged AUC for the ROC curves based on a sequence of tuning parameters. For the DINGO, ROC curve was generated based on the cutoffs of the estimated group-specific partial correlations and the differential score of the differential network.

As to the estimation of the underlying precision matrices from the simulated data matrices of *Ω*_1_ and *Ω*_2_, Table 1 showed the mean AUC averaged over 100 replications for each combination of sample size *n*, the number of vertices *p*, the level of heterogeneity *γ* and graph structure. The AUCs in our method were much larger than those in the DINGO and JGL, suggesting that our method had more accurate estimations of the conditional dependencies than the DINGO and JGL methods. When *n*, *p* and *γ* were fixed, AUC in our method was smallest in scale-free graph among three graph structures, but still large enough to demonstrate the accuracy of the estimations.

For the differential network structure, our method performed much better than the DINGO method. Note that in the paper of DINGO^14^, it only focused on the performance in the estimating the group-specific component and didn’t examine the performance of the differential scores proposed for determining the edges in the differential network. Here in our simulation, it showed that the differential scores performed poorly (Table 1). Besides, it was estimated that DINGO would take more than 50 hours as *p* > 2000 (using a Linux server with a 2.67 GHz Intel processor and 96GB of RAM). The computation time in DINGO increased exponentially as *p* increases. In step 2 of DINGO the group-specific component was estimated using expectation–maximization (EM) algorithm and in step 3 of DINGO differential scores were calculated from the bootstrap procedure. Both are computationally intensive. Therefore, it was impractical for DINGO to deal with genome-wide data which normally have the number of genes *p* > 10,000. In contrast, our test statistics, which was directly used to test the differential network structure, performed much faster than DINGO (Supplementary Fig S2). In particular, our method is still feasible even number of genes *p* = 8000.

### Real data analysis

Lung cancer is the leading cause of cancer death worldwide and adenocarcinoma is its most common histological subtype^20^. Here, we applied our algorithm to perform genome-scale differential network analysis for lung adenocarcinoma, aiming to find important molecular implications for lung cancer treatment. Gene expression profiling of 58 lung adenocarcinoma tumors and their matched histologically normal lung tissue samples were analyzed using Illumina HumanWG-6 v3.0 expression beadchip. We downloaded the normalized data from the NCBI GEO database, GSE32863^21^. Statistical analyses were limited to probes retained after applying the following filters: non-detectable expression in ≥90% of samples using a detection P-value cut-off of 0.01. We averaged the expression values of multiple probes matched to the same genes in each sample. After these data pre-processing, 7827 genes were remained for the subsequent analyses. First, we inferred the gene network by estimating the corresponding GGMs of genes for lung adenocarcinoma tumors and lung normal tissues, respectively. Then, we performed the differential network analysis by investigating the difference between two precision matrices. Table 2 listed the top 10 most significant pairs of genes in the differential network analysis. For each pair of genes in Table 2, we showed the corresponding element in the precision matrix and p-value in case and control group, respectively, as well as the corresponding partial correlation and p-values. Note that many genes have been reported previously relevant to human cancers. For example, GRN is a potent mitogen and growth factor implicated in many human cancers^22^. A somatic *RRAS* mutation (p.Gln87Leu substitution) had previously been reported as a rare somatic event in lung carcinoma^23^. Most gene pairs with very significantly strong partial correlations in control group didn’t have significant partial correlation in case group. It indicated that the connection of genes in disease samples was weaker than that in healthy samples. It was interesting to point out that there were two special pairs, LOC284230 ~RPL23 and GDI2~LOC651816. Both pairs had significant partial correlation in both case and control, however, the direction of partial correlation in case was opposite to that in control. RPL23, a tumor metastasis-related gene, was found to induce high invasiveness of a human lung adenocarcinoma cell line^24^. GDI2 overexpression reduced lung metastasis^25^.

**Table 2:** Top 10 most significant pairs of genes in the differential network analysis from the lung cancer study.

Gene	Gene	${\hat{\omega }}_{ij,1}$	p value for ${\hat{\omega }}_{ij,1}$	${\hat{\gamma }}_{ij,1}$	p value for ${\hat{\gamma }}_{ij,1}$	${\hat{\omega }}_{ij,2}$	p value for ${\hat{\omega }}_{ij,2}$	${\hat{\gamma }}_{ij,2}$	p value for ${\hat{\gamma }}_{ij,2}$	*W*	p value
GRN	TSPO	−1.220	1.603E-01	0.197	1.366E-01	−58.002	7.369E-06	0.781	2.287E-48	6.224	4.837E-10
CARHSP1	RRAS	−0.776	2.804E-01	0.150	2.642E-01	−89.694	2.145E-05	0.719	2.539E-27	6.163	7.148E-10
COMMD5	DLC1	0.399	4.554E-01	−0.103	4.481E-01	76.557	2.567E-05	−0.709	3.822E-25	−6.130	8.791E-10
CIP29	ZCCHC17	2.551	1.144E-01	−0.222	8.888E-02	−78.504	1.450E-05	0.741	3.985E-33	5.941	2.833E-09
CUTA	MRPS24	−1.061	3.074E-01	0.142	2.929E-01	−115.204	2.623E-05	0.707	6.723E-25	5.920	3.223E-09
LOC284230	RPL23	−10.744	1.777E-03	0.475	7.822E-06	−174.241	3.781E-06	0.822	4.563E-76	5.913	3.357E-09
CRBN	SERINC3	6.459	1.393E-02	−0.359	2.709E-03	−22.954	8.653E-05	0.640	2.793E-15	5.872	4.299E-09
HTRA2	RPL7L1	−0.724	6.184E-01	0.069	6.159E-01	201.324	5.377E-05	−0.667	2.353E-18	−5.814	6.089E-09
PCTK3	SUSD1	−0.351	6.442E-01	0.064	6.422E-01	−51.450	2.156E-05	0.719	2.925E-27	5.808	6.333E-09
GDI2	LOC651816	−12.125	4.843E-03	0.420	2.084E-04	39.698	2.959E-04	−0.573	5.365E-10	−5.802	6.538E-09

Note: ${\hat{\omega }}_{ij,d}$, d = 1, 2, for case and control group, respectively.

As a comparison, we also applied DiffCoEx, a method for identifying marginal correlation-based pattern changes, which builds on the commonly used Weighted Gene Coexpression Network Analysis (WGCNA) framework for coexpression analysis^2^. We selected the default parameters, such as the soft power and minimum module size in network construction and module detection. In total, 19 modules were detected, shown in the Supplementary Fig S3. Based on our proposed procedures, at a false discovery rate level of 0.05, it resulted in a differential network with 3503 nodes and 2550 edges, which consisted of 50 clusters, which had the maximal connected sub-networks with number of nodes ≥10.

**Figure 1:**
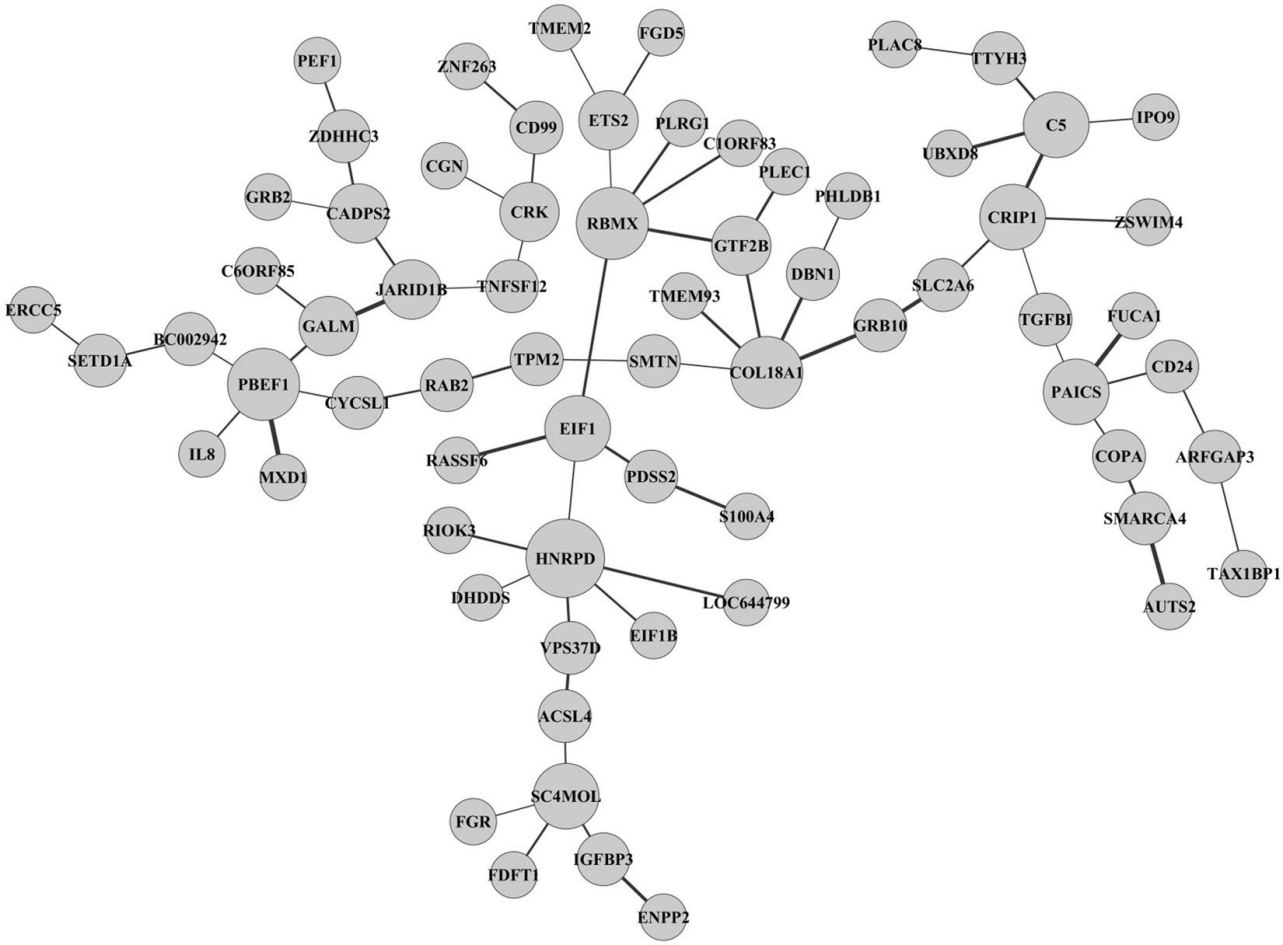
One cluster in the differential network between lung adenocarcinoma tumors and healthy samples. The sizes of nodes are proportional to their degrees. The widths of the edges are proportional to the *W* statistics.

From this differential network, we can directly know the network structure. For DiffCoEx method, however, we cannot derive any detail of the network structure within each module. It is known that analyzing network differences of gene networks between the disease and healthy conditions could be helpful for understanding the genetic mechanisms of the disease. In terms of differential network structure, our method is much better for us to understand the mechanism of the disease. By using the partial correlation approach, in contrast, our differential network analysis resulted in much sparser network. As the partial correlation quantified the correlation between two genes after controlling other genes’ effects, which provided with useful information to distinguish the causal correlations in the network. Functional analysis for the differential network showed that the top significantly enriched KEGG pathways were “Metabolic pathways” (hypergeometric test, *p* value = 6.43e–63) and “Pathways in cancer” (p value = 1.59e–24). The full results of functional enrichment analysis of the differential network and clusters from DiffCoEx were shown in the Supplementary Tables 1 and 2, respectively, to illustrate the functional compositions of two methods. For simple illustration and visualization, we showed a large cluster from the differential network in the Fig. 1.

## Discussion

Motivated by an important biological question that how the network structure of cellular interactome change from one condition to another, we derived a formal statistical test for the differential network analysis based on the inference of GGM. Our method not only provided statistical inference of the difference of edge strength between graph nodes in the differential network analysis, but also a multiple testing procedure for simultaneously testing the large number of tests with FDR control to infer the structure of the differential network. The source code of the implementation is available at Supplementary File 1.

The hypothesis testing of differential network was directly based on estimator of ω_ij,1_ − ω_ij,2_. First, we implemented the asymptotically normal and efficient estimation of GGM. Then we performed the hypothesis testing of differential network by comparing the difference between two precision matrices from two conditions, respectively. Moreover, we performed a multiple testing procedure with FDR control for simultaneously testing *p*(*p* − 1)/2 hypotheses. The procedure for the differential network analysis we present here had the advantage in a global and unbiased manner.

First, our method provided with a rigorous statistical test for the difference of conditional dependence between two different conditions. It represented a major improvement over earlier procedure, which built two global gene networks for the disease and healthy samples respectively under the GGM framework with a FDR threshold for determining the existence of edges, and then compared the topological changes with the unique edges that belonged to only one of the networks^9^. Our method directly resulted in the differential network structure with statistical estimation and inference of the difference of edge strength between graph nodes. Second, we adopted a multiple testing procedure for simultaneously testing the large number of tests with FDR control to infer the structure of the differential network, as the standard Bonferroni or naive FDR corrections would lose power. For the multiple testing problem, we proposed to threshold test statistics directly rather than using p-values as in Benjamini and Hochberg (BH)^26^, mainly because the BH method for controlling FDR required the independence between p-values, while our test statistics may be weakly dependent of each other, which is natural in GGM estimation^17^. Third, through the realistic simulation studies with different combinations parameters of sample size, number of vertices, level of heterogeneity and graph structure, we demonstrated that our method performed much better than the current available methods in terms of accuracy and computational time. Then we applied it on a real data set and successfully constructed the differential network for lung adenocarcinoma. The differential network analysis can help reveal how the architecture of gene network is rewired during a cellular or adaptive response and elucidate fundamental molecular mechanism of biological processes. Especially for cancer research, our method will be very helpful for identifying novel driver genes or pathways. In our real data analysis on the lung adenocarcinoma, we revealed a differential network with 3503 nodes and 2550 edges, which consisted of 50 clusters with a FDR threshold at 0.05. Especially, for the top gene pairs in the differential analysis, many of them have been reported relevant to human cancers. Our method can be a powerful tool of network analysis based on GGM, especially for high-dimensional biological data.

However, there were several limitations of our method. First, the inference of GGM relies on the Gaussian assumption on the data. Nowadays, high-throughput RNA sequencing (RNA-seq) is the standard tool for gene expression analysis. Analyzing RNA-seq data depends on estimates of read count variability, which are statistically modeled as the negative binomial distribution^27^. Second, currently our method can only be applied to the study of differential network analysis between two conditions. We will extend our differential network approach for sequence data as well as multiple conditions in future studies.

## Conclusion

In summary, we derived a formal statistical test for the differential network analysis based on the inference of GGM, as well as a multiple testing procedure for simultaneously testing the large number of tests with FDR control to infer the structure of the differential network. Through simulation studies, we demonstrated that our method performed much better than the current available methods in terms of accuracy and computational time. Our method will be very helpful in differential network analysis for identifying novel driver genes or pathways in high-dimensional biological data.

## Supplementary information

Supplementary Info

Supplementary file 1
