# Supplementary material for: A Statistical Test for Differential Network Analysis Based on Inference of Gaussian Graphical Model
Source: Sci Rep. 2019 Jul 26;9:10863. doi: 10.1038/s41598-019-47362-7 (PMC6659630; doi:10.1038/s41598-019-47362-7)
Supplement: Supplementary file 1 — Supplementary Info [file 41598_2019_47362_MOESM1_ESM.docx]

**Title page**

# A Statistical Test for Differential Network Analysis Based on Inference of Gaussian Graphical Model

**Authors**

Hao He^1^, Shaolong Cao^1,2^, Ji-gang Zhang^1^, Hui Shen^1^, Yu-Ping Wang^1,2^, Hong-wen Deng^1*^

**Affiliations**

^1^Center for Bioinformatics and Genomics, Department of Global Biostatistics and Data Science, Tulane University School of Public Health and Tropical Medicine, New Orleans, LA 70112, USA

^2^Department of Biomedical Engineering, Tulane University, New Orleans, LA, 70118, USA

***Corresponding author**

Hong-Wen Deng, Ph.D.

Professor, Center for Bioinformatics and Genomics,

Department of Global Biostatistics and Data Science,

School of Public Health and Tropical Medicine, Tulane University,

1440 Canal St., Suite 2001, New Orleans, LA 70112, USA,

Email: [hdeng2@tulane.edu](mailto:hdeng2@tulane.edu)

Supplementary Table 1. The result of function analysis for the differential network

| Name | #Gene | PValue |
| --- | --- | --- |
| Metabolic reprogramming in colon cancer | 11 | 0.00459 |
| Photodynamic therapy-induced HIF-1 survival signaling | 9 | 0.0195 |
| Pancreatic adenocarcinoma pathway | 17 | 0.0196 |
| EPO Receptor Signaling | 7 | 0.0223 |
| Apoptosis | 16 | 0.0239 |
| ATM Signaling Network in Development and Disease | 10 | 0.0261 |
| PDGFR-beta pathway | 7 | 0.0394 |
| Target Of Rapamycin (TOR) Signaling | 8 | 0.0454 |
| 4-hydroxytamoxifen, Dexamethasone, and Retinoic Acids Regulation of p27 Expression | 5 | 0.0454 |
| Endometrial cancer | 12 | 0.0476 |

Supplementary Table 2. The result of function analysis for the clusters from DiffCoEx

| Module name | Term | #Gene | Pvalue |
| --- | --- | --- | --- |
| darkgrey | Oxidative phosphorylation | 27 | 4.06E-14 |
| grey | Lysosome | 52 | 5.72E-11 |
| blue | Ribosome | 48 | 9.36E-11 |
| magenta | Pertussis | 8 | 1.22E-08 |
| orange | Ribosome | 13 | 2.39E-07 |
| red | Focal adhesion | 8 | 9.48E-05 |
| yellow | Metabolic pathways | 12 | 0.000133 |
| darkorange | Pathogenic Escherichia coli infection | 5 | 0.000157 |
| white | TNF signaling pathway | 3 | 0.00059 |
| saddlebrown | RNA degradation | 2 | 0.004825 |
| steelblue | Ubiquitin mediated proteolysis | 2 | 0.011649 |
| darkgreen | RNA degradation | 2 | 0.01193 |
| royalblue | Pyrimidine metabolism | 2 | 0.022379 |


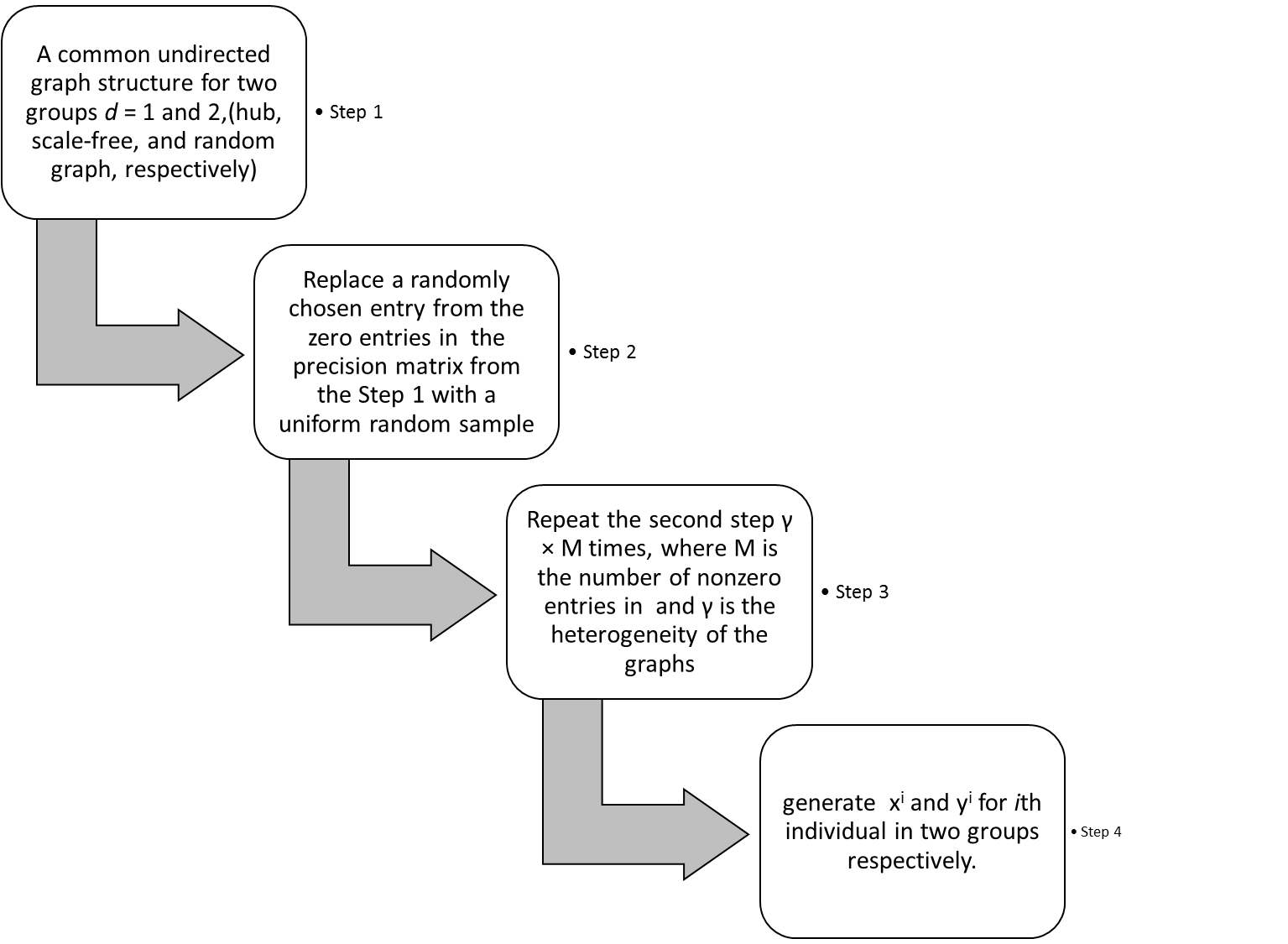
Supplementary Fig S1. A workflow of simulation steps.


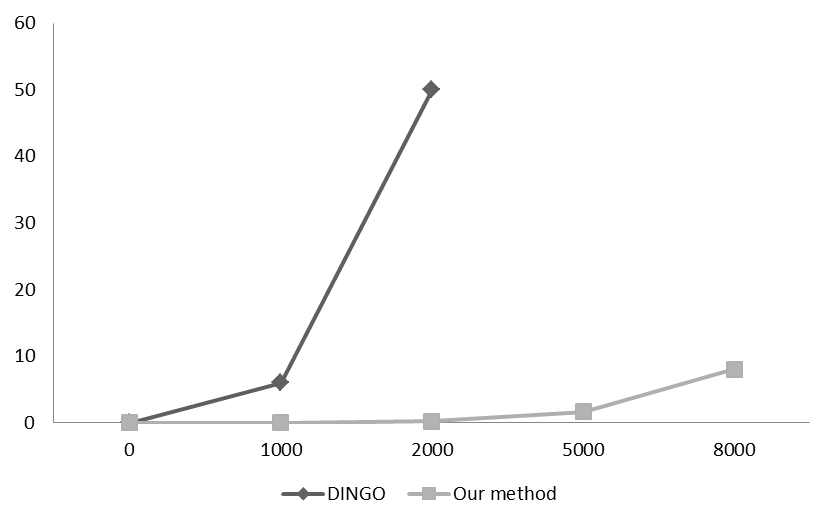


***p***

***Hours***

Supplementary Fig S2. Comparison of computational time.


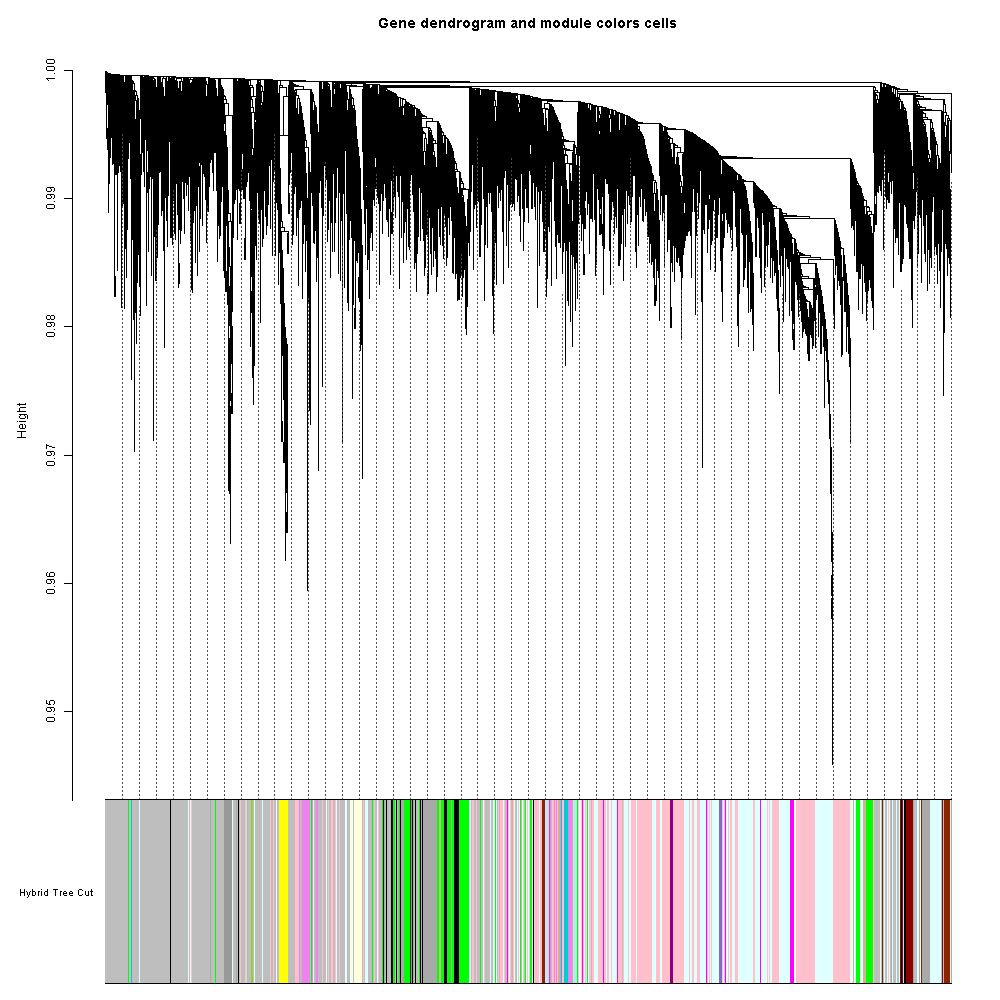


Supplementary Fig S3. Clustering dendrogram and module assignment by DiffCoEx. Topological overlap dissimilarity measure is clustered by average linkage hierarchical clustering and module assignments (dynamic hybrid algorithm) are denoted in the color bar (bottom). 7827 genes were assigned to 19 modules.
